# Construction of a Diagnostic Model and a lncRNA-Associated ceRNA Network Based on Apoptosis-Related Genes for Schizophrenia

**DOI:** 10.1155/2023/7017106

**Published:** 2023-06-20

**Authors:** Zi-long Ma, Run-lan Wang, Lili Meng

**Affiliations:** ^1^Department of Psychiatry, Wuhan Mental Health Center, Wuhan, Hubei Province 430012, China; ^2^Department of Sleep, Wuhan Hospital of Psychotherapy, Wuhan, Hubei Province, China

## Abstract

**Methods:**

Gene expression profiles and apoptosis-related data were downloaded from the Gene Expression Omnibus and Molecular Signature databases, respectively. Apoptosis-related differentially expressed mRNAs (DEGs) and miRNAs (DEMs) from blood samples between the schizophrenia and healthy control individuals were screened. A diagnostic model was developed using the data from univariate and least absolute shrinkage and selection operator (LASSO) regression analyses, followed by validation using the GSE38485 dataset. Cases were divided into low-risk (LR) and high-risk (HR) groups based on the risk score of the model, and differences in immune gene sets and pathways between these two groups were compared. Finally, a ceRNA network was constructed by integrating long non-coding RNAs (lncRNAs), DEMs, and DEGs.

**Results:**

A diagnostic model containing 15 apoptosis-related genes was developed and its diagnostic efficiency was found to be robust. The HR group was correlated with higher immune scores of chemokines, cytokines, and interleukins; it was also significantly involved in pathways such as pancreatic beta cells and early estrogen response. A ceRNA network composed of 2 lncRNAs, 14 miRNAs, and 5 mRNAs was established.

**Conclusions:**

The established model is a potential tool to improve the diagnostic efficiency of patients with schizophrenia, and the nodes included in the ceRNA network might serve as biomarkers and therapeutic targets for schizophrenia.

## 1. Introduction

Schizophrenia is a complex neuropsychiatric syndrome that affects approximately 1% of the world's population and poses a serious health burden [1]. Symptoms of schizophrenia include psychotic episodes and cognitive dysfunction, resulting in a lack of motivation and social withdrawal [2]. Patients with schizophrenia develop mental illness and disability, which may ultimately lead to disturbances in their daily lives and a reduced life expectancy [3]. The heterogeneous phenotypic and genetic characteristics of schizophrenia continue to present challenges for exploring its etiology, diagnosis, and developing treatment protocols.

Increasing attention is being paid to the study of environmental factors associated with alterations in gene expression through epigenetic regulation [4]. Moreover, discoveries in the field of schizophrenia pathophysiology have made it possible to establish reliable biomarkers [5]. It has been reported that effective biomarkers can verify potential therapeutic targets or predict responses and can inform clinical diagnoses or treatment strategies for schizophrenia [6]. Therefore, the investigation of potential biomarkers of schizophrenia can help develop diagnostic tools.

Apoptosis is a regulated form of cell death that remains active during neurodevelopment; it can also be reactivated under a variety of neuropathological conditions [7]. Accumulating evidence has demonstrated that apoptosis plays a potential role in the pathophysiology of schizophrenia. Proapoptotic triggers can lead to non-lethal apoptotic activity, which may induce neuronal and synaptic elimination without cell death, resulting in cognitive decline in patients with schizophrenia [8, 9]. Additionally, DNA fragmentation, a feature of apoptosis, has been observed in the cortical regions of schizophrenia patients [10]. Nevertheless, there is no report about research on apoptosis-related biomarkers in the pathogenesis of schizophrenia.

In addition to mRNA, non-coding RNAs, such as lncRNAs, may be also involved in the pathogenesis of schizophrenia. A high expression of lncRNAs in the brain contributes to the healthy function of neurons and synapses; these molecular pathways are frequently found to be dysfunctional in schizophrenia [11]. Specific changes in lncRNAs and miRNAs have been observed in the brains with schizophrenia, further supporting their roles in this disorder [12]. In addition, the competing endogenous RNA (ceRNA) network composed of miRNAs, mRNA, and lncRNAs plays a key role in maintaining synaptic density and neurogenesis, which is also implicated in schizophrenia [13]. However, there have been few studies on the construction of ceRNA networks based on apoptosis-related genes. In this study, we downloaded the gene expression profile of blood samples from schizophrenia and normal control (NC) cases from several public databases, followed by the identification of differentially expressed genes (DEGs) and miRNAs (DEMs). Apoptosis-related genes were extracted from the public database and integrated with DEGs to construct a diagnostic model of schizophrenia. A ceRNA network was established to reveal the regulatory mechanisms of schizophrenia. These findings may help elucidate the disease mechanisms, improve diagnosis, and guide the development of new drugs.

## 2. Materials and Methods

### 2.1. Data Acquirement

A total of 310 schizophrenia and 242 NC samples obtained from four public datasets were included in this study. In brief, mRNA-seq expression profiles of the 341 samples (182 schizophrenia and 159 controls) were downloaded from the Gene Expression Omnibus (GEO) database (https://www.ncbi.nlm.nih.gov/geo/), and the miRNA expression profiles of 211 samples (128 schizophrenia and 83 controls) were extracted from the ARRAYEXPRES database (https://www.ebi.ac.uk/arrayexpress/). Detailed information for each dataset is presented in Table 1.

### 2.2. Data Preprocessing

After data standardization, the probe number was matched with the gene symbol using the downloaded platform annotation file of the GEO datasets. According to the source of the samples, data from peripheral blood mononuclear cells (PBMCs) were merged as the training sets (GSE27383 and GSE54913), and those from whole blood were merged as the validation set (GSE38485). The R package inSilicoMerging (v1.14.0) [14] was used to merge datasets, after which the empirical Bayes method [15] was applied to remove batch effects, and the obtained matrix was used for subsequent analysis.

Based on the miRNA microarray data from the ARRAYEXPRESS database, the probe number was matched with miRNA using the downloaded platform annotation file to obtain the expression value of miRNA.

### 2.3. Identification of Apoptosis-Related DEGs

A total of 228 apoptosis-related genes were downloaded from two datasets (HALLMARK_APOPTOSIS and KEGG_APOPTOSIS) hosted on the Molecular Signature (MSigDB) database (v7.1). Next, these genes were mapped to the obtained mRNA matrix, and 189 apoptosis-related genes were screened for further analysis. Differential expression analysis of schizophrenia vs. controls was performed according to the expression level of these genes established using the Student's *t* test. Genes with *p* < 0.05 were considered apoptosis-related DEGs.

### 2.4. Correlation and Protein–Protein Interaction Analyses

To understand the relationship between genes, the Pearson correlation coefficient (PCC) between any two apoptosis-related DEGs was calculated. Additionally, these genes were entered into the STRING database, and the parameters were set to human and medium confidence (protein–protein interaction [PPI] score = 4) to obtain PPI pairs. After obtaining PPI pairs, the PPI network was visualized using the Cytoscape software (v3.6.1) [16].

### 2.5. Construction of a Diagnostic Model

Univariate logistic regression was performed to identify disease-associated genes and genes with *p* < 0.05. least absolute shrinkage and selection operator (LASSO) Cox regression with 20-fold cross-validation was conducted to obtain the optimal gene signature and corresponding coefficients using the glmnet package in R (v4.0–2) [17]. The risk score (RS) for each patient was calculated using the following formula: *RS* = ∑*β*_gene_ × Exp_gene_; where *β*_gene_ represents the LASSO coefficient, and Exp_gene_ represents the expression level of the gene. Based on the median score, the samples were assigned to low-risk (LR) and high-risk (HR) groups. To verify whether this model was effective for whole blood, we used the same formula to construct a diagnostic model for the validation set. In addition, a receiver operating characteristic (ROC) curve was plotted to determine the predictive performance of the model.

### 2.6. Correlation Analysis of Immune and Different Risk Groups

Immune-related gene sets were obtained from the ImmPort database [18], and the immune gene set score for each sample was calculated using the single-sample Gene Set Enrichment Analysis (ssGSEA) algorithm in the gene set variation analysis (GSVA). The difference in immune scores between the HR and LR groups was assessed using a *t* test. PCC between immune gene sets and each gene in the model was calculated and displayed using a heatmap. Moreover, differences in human leukocyte antigen (HLA) family genes between the two risk groups were assessed, followed by a calculation of the PCC between HLA family genes and each gene in the model.

### 2.7. Comparative Analysis of Pathway between HR and LR Groups

Based on the “h.all.v7.4. symbols.gmt” in the MSigDB database as an enrichment background, the GSVA score of each HALLMARK pathway in samples was calculated using the GSVA algorithm [19]. Differences in pathways between the LR and HR groups were analyzed using the limma package in R (v3.10.3) [20]. Pathways with |t score| > 1 and adjusted *p* < 0.05 were regarded as significantly enriched.

### 2.8. Analysis of ceRNA Regulatory Mechanism

DEMs between schizophrenia and controls were identified using the Student's *t* test, and miRNAs with *p* < 0.05 were considered to be differentially expressed. The miRWalk 3.0 was used to perform the miRNA prediction of genes in the diagnostic model, with default parameters: binding probability > 95%, binding site position = 3UTR. The predicted miRNAs were then intersected with DEMs, and the overlapping miRNAs were obtained to generate miRNA–mRNA pairs. Furthermore, DIANA-LncBase was used to predict lncRNAs targeted by miRNAs in miRNA–mRNA pairs. These lncRNAs were then matched with the DEGs within schizophrenia vs. controls to obtain the differentially expressed lncRNAs and the corresponding lncRNA–miRNA pairs. Finally, the mRNAs and lncRNAs regulated by the same miRNA were screened, and a ceRNA network based on the mRNA–miRNA–lncRNA pair was constructed using the Cytoscape software.

## 3. Results

### 3.1. Data Preprocessing

**Figure 1:**
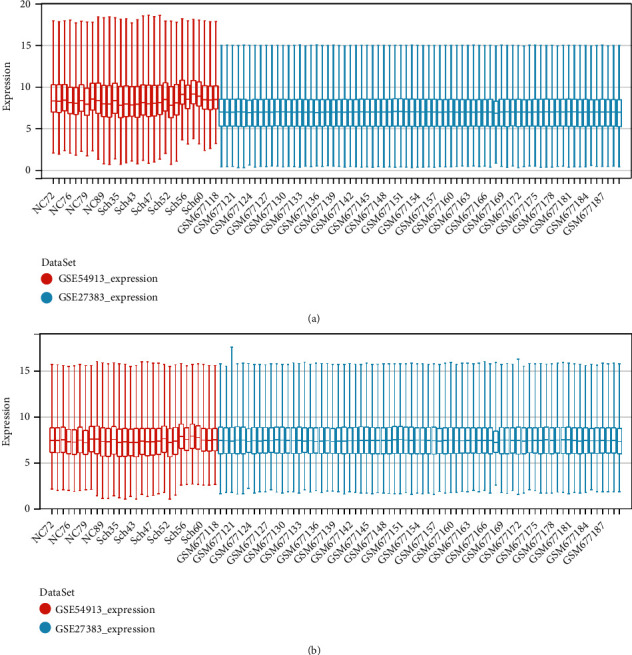
Box plot of data distribution before (a) and after (b) the removal of batch effects. The red boxes represent the normal control samples, and the blue boxes represent the schizophrenia sample.

As described in Materials and Methods section, the data were merged, and batch effects were removed. The distribution of data after removing the batch effect tended to be consistent with the median in a line, indicating that our data were of high quality and could be used for subsequent analyses (Figures 1(a) and 1(b)). In total, 61 schizophrenia and 41 control samples were included in this analysis, and the expression values of 10,810 genes were obtained.

### 3.2. Apoptosis-Related DEGs in Schizophrenia

**Figure 2:**
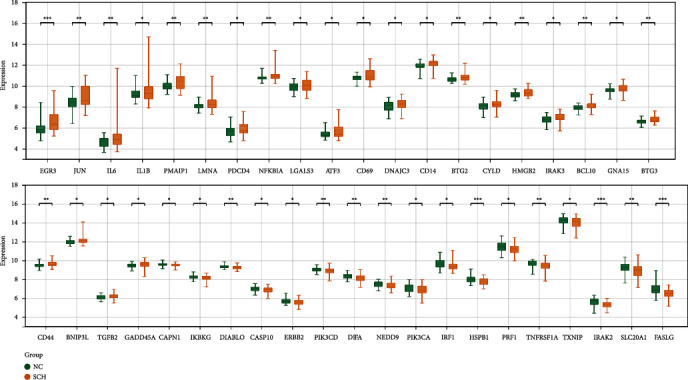
Apoptosis-related differentially expressed genes (DEGs) between schizophrenia (SCZ) and normal control (NC) samples.

After integrating the above genes and apoptosis-related genes, 189 genes were screened for further analyses. The expression values of these genes in each sample were extracted, and differential analysis was performed. As per the set threshold (*p* < 0.05), 41 apoptosis-related DEGs were identified between the schizophrenia and control groups (Figure 2).

### 3.3. Correlation Analysis and PPI Network Construction

**Figure 3:**
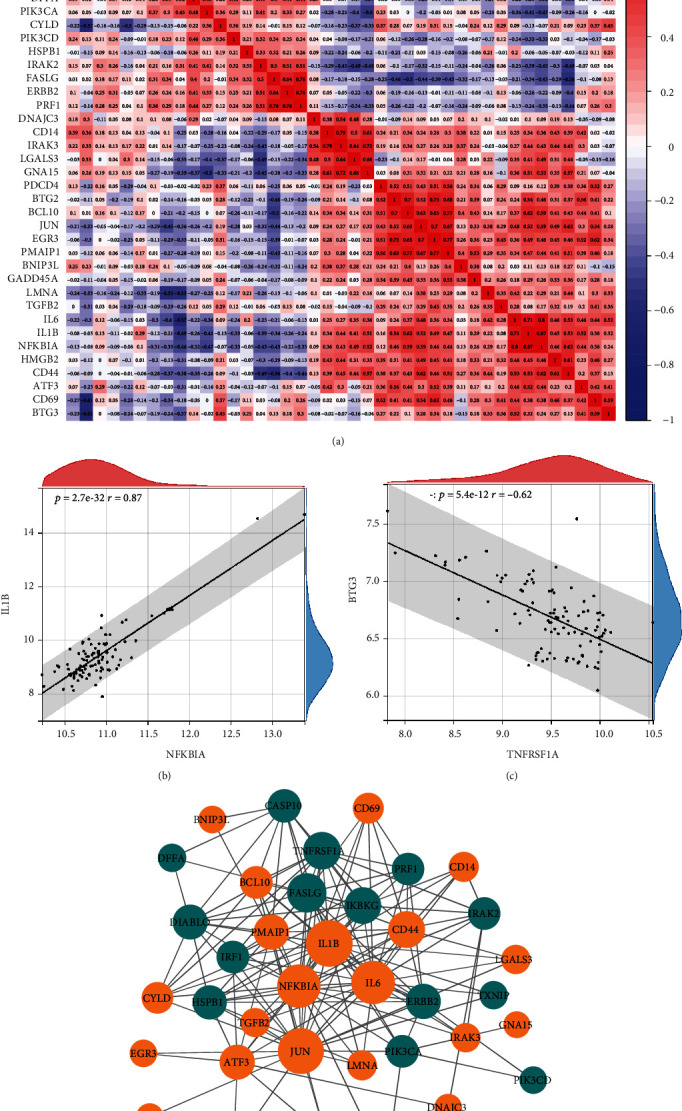
Correlation analysis and construction of PPI network. (a) Heatmap revealing the correlation of apoptosis-related DEGs. (b) *NFKBIA* is positively correlated with *IL1B*. (c) *TNFRSF1A* is negatively correlated with *BTG3*. (d) PPI network of 37 genes. Green nodes represent downregulated genes, and the orange nodes represent upregulated genes. The size of the node represents the degree score between genes.

The PCCs between two apoptosis-related genes were calculated to investigate the relationship between these DEGs. The expression of these genes was closely correlated (Figure 3(a)). *NFKBIA* and *IL1B* had the strongest positive correlation (Figure 3(b)), whereas *TNFRSF1A* and *BTG3* had the strongest negative correlation (Figure 3(c)). The interaction between these genes was predicted using the STRING database, and a PPI network containing 156 edges and 37 nodes was constructed (Figure 3(d)). Genes such as *IL1B*, *JUN*, *IL6*, *NFKBIA*, and *FASLG* were simultaneously linked to multiple nodes in the network and may be considered key genes.

### 3.4. Establishment and Validation of the 15-Gene Signature Diagnostic Model

**Figure 4:**
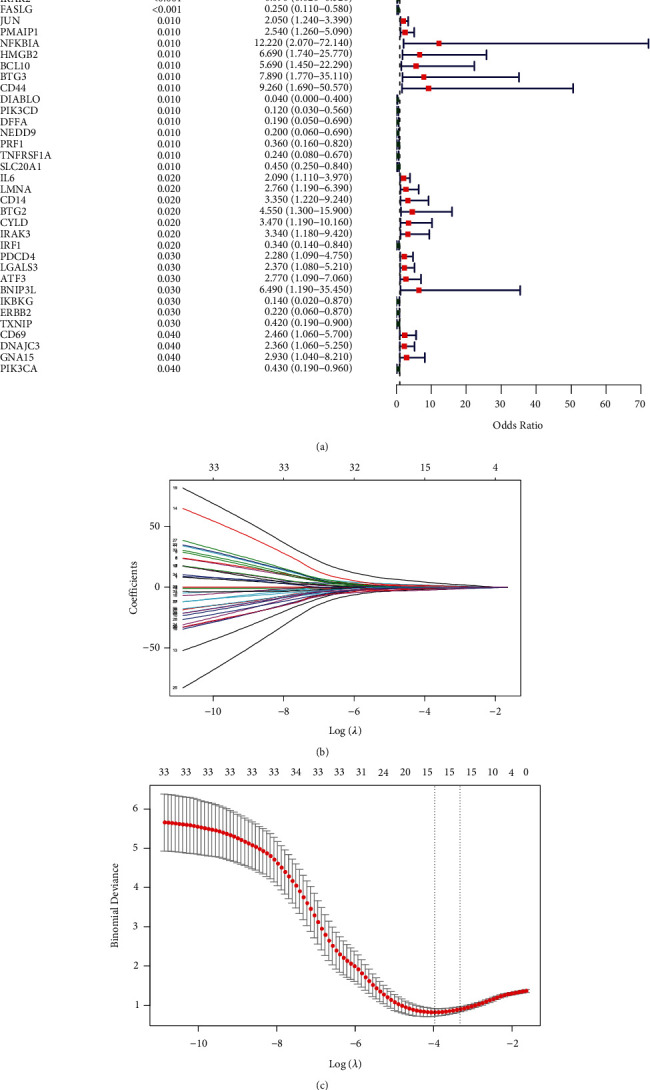
Construction of a diagnostic model in patients with schizophrenia. (a) Forest plot of the diagnostic effect of 36 genes (*p* < 0.05) via univariate logistic regression analysis. (b) LASSO coefficient profiles of 15 screened DEGs. (c) Twenty folds cross-validation for LASSO analysis.

After the univariate logistic regression analysis, 36 genes (*p* < 0.05) were screened and regarded as disease-related genes (Figure 4(a)). Then, 15 optimal variables were selected from 36 diagnostic genes using the LASSO regression analysis, which was used to construct a diagnostic model (Figures 4(b) and 4(c)). The diagnostic RS was calculated using the following formula: RS = (0.025 × *EGR*3) + (0.292 × *PMAIP*1) + (0.639 × *LGALS*3) + (1.665 × *DNAJC*3) + (0.352 × *CD*14) + (1.222 × *CYLD*) + (4.053 × *BTG*3) − (0.606 × *DIABLO*) − (1.852 × *PIK*3*CD*) − (1.516 × *DFFA*) − (0.111 × *PIK*3*CA*) − (1.103 × *IRF*1) − (2.532 × *HSPB*1) − (0.792 × *TNFRSF*1*A*) − (0.896 × *FASLG*).

**Figure 5:**
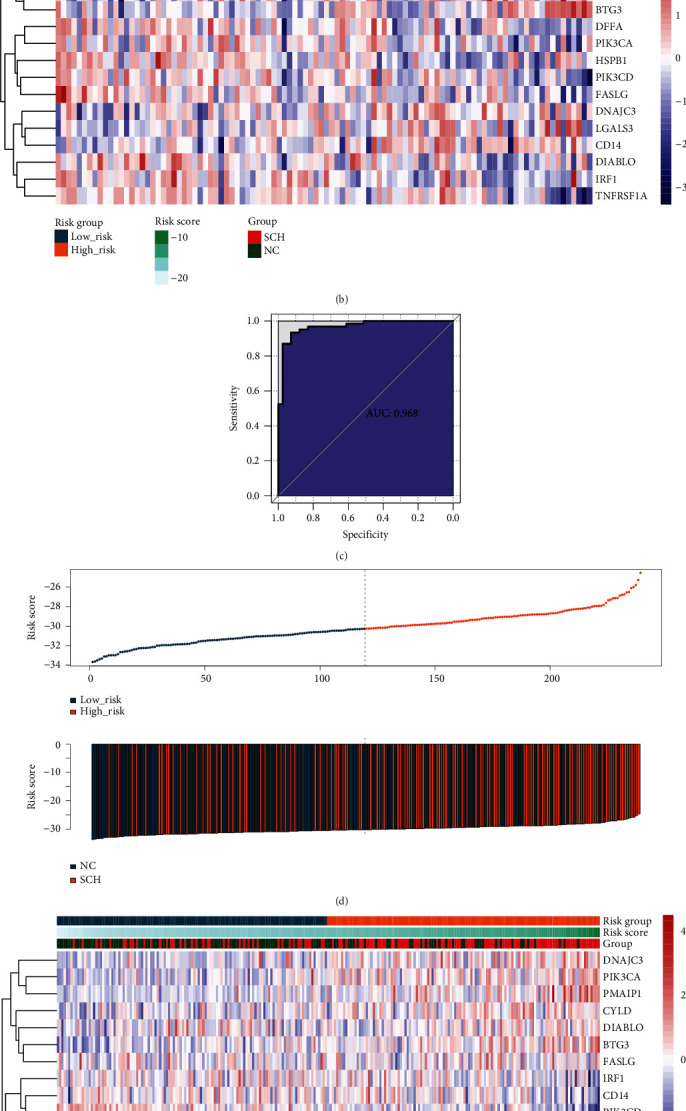
Evaluation of the diagnostic performance of diagnostic model in the training and validation sets. (a) Risk score (RS) distribution in samples classified as low-risk (LR) and high-risk (HR) groups in the training set. (b) Heatmap of the expression level of apoptosis-related gene signature in the training set. (c) Receiver operating characteristic (ROC) analysis of the diagnostic model using the training set. (d) RS distribution for the LR and HR groups in the validation set. (e) Heatmap of 15 apoptosis-related genes expression in the validation set. (f) ROC analysis of the diagnostic model using the validation set.

To evaluate the reliability and robustness of this model, we performed a series of analyses on the training and validation sets. Using the median RS as the cutoff, patients in the two sets were assigned to the LR and HR groups. In the training set, the HR group contained more disease samples, whereas the LR group mainly consisted of NC samples (Figure 5(a)). The expression levels of the 15 genes in the LR and HR groups are displayed in a heatmap (Figure 5(b)). The area under the curve (AUC) value of this diagnostic model was 0.968 (Figure 5(c)). In addition, the RS distribution and gene expression heatmap for the validation set were also plotted (Figures 5(d) and 5(e)). The AUC value of this model for the validation set was 0.712 (Figure 5(f)). Taken together, these results indicated that the diagnostic performance of this model was reliable.

### 3.5. Relationship between the Immune Gene Sets and Different Risk Groups

**Figure 6:**
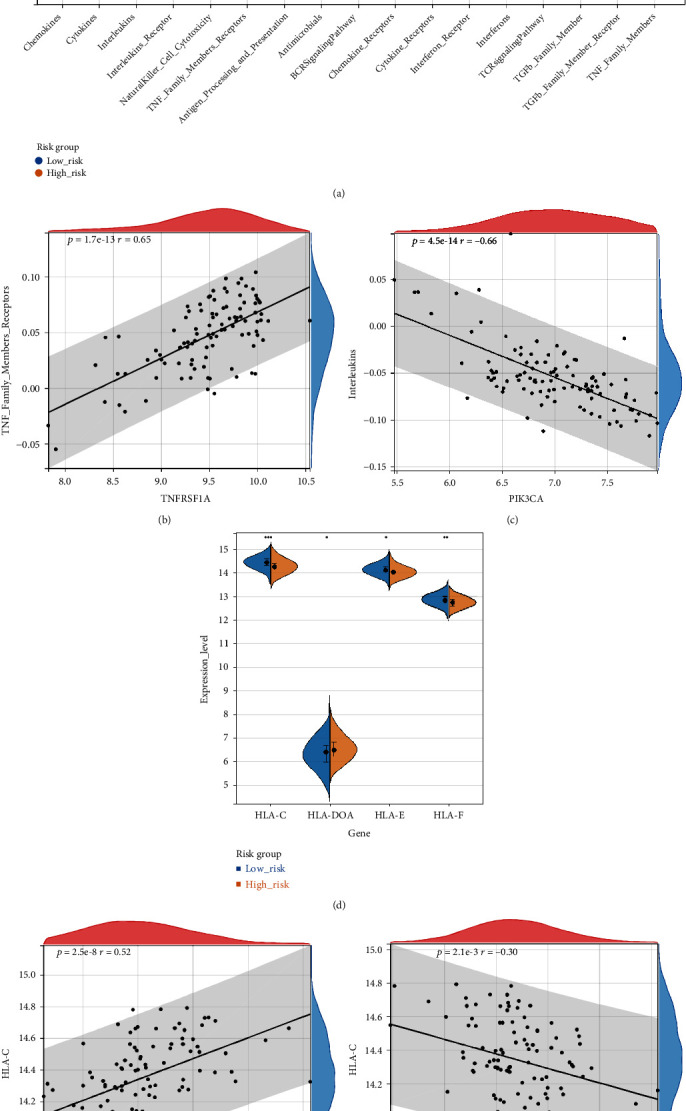
Correlation between the immune gene sets and different risk groups. (a) Differences in the immune gene sets between LR and HR groups. (b) *TNFRSF1A* showing a positive correlation with tumor necrosis factor (TNF) family member receptors. (c) *PIK3CA* showing a negative correlation with interleukins. (d) Differences in the human leukocyte antigen (HLA) family genes between LR and HR groups. (e) *IRF1* was positively associated with HLA-C. (f) *CYLD* was negatively correlated with HLA-C.

The enrichment scores of the 17 immune gene sets in each sample were calculated and compared between the LR and HR groups. The results showed that the enrichment scores of chemokines, cytokines, and interleukins were higher in the HR group than in the LR group, while the scores of the interleukin receptors, natural killer cell cytotoxicity, and tumor necrosis factor (TNF) family member receptors were higher in the LR group (Figure 6(a)). The correlation between these different gene sets and each gene in the model was also analyzed. We found a strong positive correlation between *TNFRSF1A* and TNF family member receptors (Figure 6(b)) and a strong negative correlation between *PIK3CA* and interleukins (Figure 6(c)).

Moreover, differences in the HLA gene family between LR and HR were also compared. Four genes were significantly different between the two groups; the expression level of *HLA-DOA* was increased in the HR group, whereas those of *HLA-C*, *HLA-E*, and *HLA-F* were decreased in the HR group (Figure 6(d)). The correlation analysis indicated that *IRF1* had a strong positive correlation with *HLA-C* (Figure 6(e)) and *CYLD* had a strong negative correlation with *HLA-C* (Figure 6(f)).

### 3.6. GSVA of LR and HR Groups

**Figure 7:**
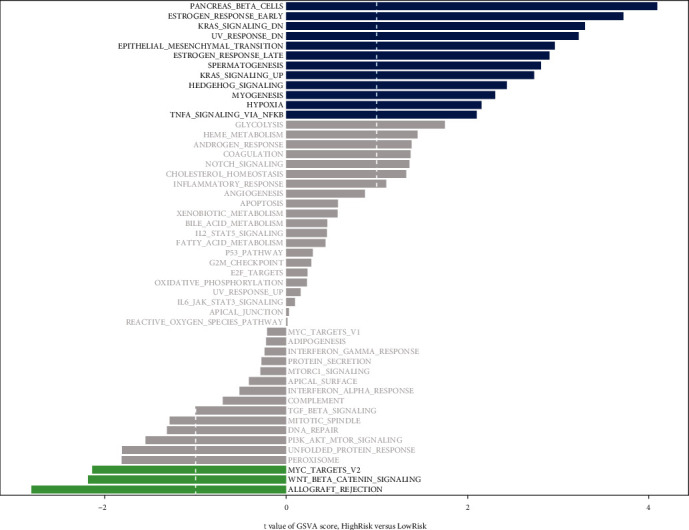
GSVA analysis of LR and HR groups. The blue bars represent pathways significantly enriched in the HR group, and the green bars represent pathways significantly enriched in the LR group.

GSVA was performed to explore the differences in pathways between the HR and LR groups. The results revealed that 12 pathways were significantly enriched in the HR groups, including pancreatic beta cells, early estrogen response, and kras signaling. Meanwhile, the LR group was significantly involved in three pathways, including MYC target v2, WNT beta-catenin signaling, and allograft rejection (Figure 7).

### 3.7. Construction of lncRNA-Related ceRNA Network

**Figure 8:**
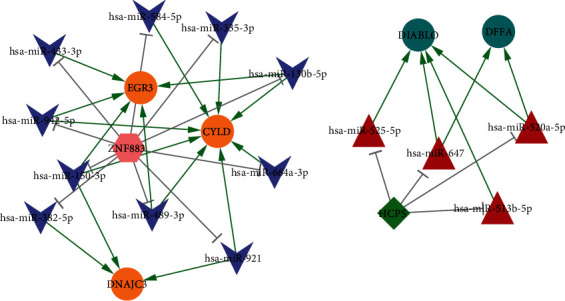
LncRNA-related ceRNA network. Orange and malachite green circles represent upregulated and downregulated mRNA, respectively. Red triangles and purple arrowheads represent upregulated and downregulated miRNAs, respectively. Pink hexagon and green diamond represent upregulated and downregulated lncRNAs, respectively.

A total of 103 DEMs between the schizophrenia and NC groups were screened, and miRNAs targeting 15 genes in the model were also identified. After integrating the analysis, 88 miRNA–mRNA pairs were obtained. Then, lncRNAs targeting miRNAs were predicted, and 26 lncRNA–miRNA pairs were obtained. Finally, 22 mRNA–miRNA–lncRNA pairs that met the ceRNA regulatory mechanism were selected for network construction. The ceRNA network was composed of 2 lncRNAs (*ZNF883* and *HCP5*), 14 miRNAs (miR-150-3p, miR-520a-5p, and miR-130b-5p), and 5 mRNAs (*EGR3*, *CYLD*, *DNAJC3*, *DIABLO,* and *DFFA*), with 14 lncRNA–miRNA and 22 miRNA–mRNA pairs (Figure 8).

## 4. Discussion

As an important psychiatric disorder, schizophrenia affects patients and their families by disrupting healthy functioning and thinking [21]. Large-scale transcriptomic [22], genomic [23], and epigenomic [24] studies have revealed the multifactorial biological mechanism of schizophrenia; however, its etiology remains elusive. Current anti-schizophrenia drugs only treat its symptoms and are associated with a HR of serious adverse effects [25]. In order to further understand the pathogenesis of schizophrenia and to improve its diagnosis and treatment, we constructed a diagnostic model based on apoptosis-related genes and explored the potential of ceRNA regulatory mechanisms that may be linked to the disease.

In this study, we screened apoptosis-related DEGs between schizophrenia and control samples and developed a 15-gene-based diagnostic model. Validation analysis was performed to evaluate the diagnostic performance of this model, and the results showed that the AUC values of the models in the training and validation sets were larger than 0.7 [26], indicating that this model had satisfactory diagnostic ability. Of these 15 genes, 8 have been reported to be associated with schizophrenia. *LGALS3* encodes a member of the galectin family and may play a role in the pathogenesis of schizophrenia by participating in inflammatory processes in the central nervous system; high expression levels of *LGALS3* have been detected in patients with schizophrenia [27]. The protein encoded by *CD14* is a surface antigen that may reduce or suppress severe inflammatory responses [28]. A previous study revealed that the expression level of *CD14* was significantly higher in schizophrenia samples than in controls, indicating an inflammatory state [29]. *PIK3CD* encodes phosphoinositide 3-kinases (PI3Ks) that are involved in the immune response and are associated with neurodevelopmental disorders, including schizophrenia [30]. Etemadikhah et al. [31] reported that *PIK3CD* is downregulated in schizophrenia and may be considered as a potential therapeutic target. *PIK3CA* also encodes the PI3Ks enzyme and its activity is decreased in patients with schizophrenia [32]. As a specific peripheral immune biomarker, the expression level of *IRF1* is decreased in patients with schizophrenia [33]. The overexpression of *HSPB1* directly inhibits apoptotic pathways to increase neuronal survival, thereby protecting against injury-induced nerve death [34]. Changes in *HSPB1* expression levels were observed in patients with schizophrenia, and *HSPB1* polymorphisms were associated with an increased risk of schizophrenia [35]. *TNFRSF1A* is downregulated in elderly schizophrenia subjects, which may be related to cognitive decline [36]. *FASLG* acts as a marker of apoptosis, and increased apoptotic signaling occurs at the onset of schizophrenia and is associated with treatment progression [37]. Taken together, the previously reported expression levels of these genes are consistent with our results. We speculated that these genes might be involved in schizophrenia by affecting immune-related biological processes. However, the roles of the remaining genes in the pathogenesis of schizophrenia have not yet been investigated. Previous studies have shown that genes such as *PMAIP1* [38], *DNAJC3* [39], *DFFA* [40], and *BTG3* [41] are involved in apoptosis, but their specific mechanism of action in schizophrenia needs to be further explored.

The constructed diagnostic model could categorize individuals into LR and HR groups. The immune analysis showed that the immune scores for chemokines, cytokines, and interleukins were significantly higher for patients in the HR group than those in the LR group. Chemokines are promising biomarkers of inflammation and immune activation, which may be associated with psychiatric disorders [42]. A clinical trial found that the levels of chemokines with neuroimmunomodulatory effects are higher in patients with schizophrenia, particularly in the elderly [43]. The potential applications of chemokines as diagnostic or therapeutic biomarkers should be considered in future clinical studies [44]. Moreover, several cytokines may serve as state markers for acute exacerbations or as characteristic markers of schizophrenia [45]. Together, these immune-related gene sets have been confirmed to play important roles in the development of schizophrenia, suggesting that the model established in this study has diagnostic value and research significance.

Furthermore, we constructed a ceRNA network based on 5 mRNAs, 14 miRNAs, and 2 lncRNAs. Among these, two lncRNAs, *ZNF883* and *HCP5*, may play a role in the pathogenesis of schizophrenia. Gong et al. recently found that ZNF883 is upregulated in hippocampal neurons and could be considered as a biomarker and therapeutic target for epilepsy [46]. *HCP5* is mainly detected in immune organs such as the blood, spleen, and thymus [47]. *HCP5* can be used as an immune-related marker for various human malignancies [48, 49]; however, its role in schizophrenia has not been elucidated. Meanwhile, miRNAs, such as miR-150-3p, miR-520a-5p, and miR-130b-5p, were linked to at least two mRNAs. These miRNAs are involved in immunological dysfunction; however, their roles in schizophrenia have not been reported. According to the present study, we speculate that these genes might contribute to the disease by affecting the immune response.

It should be noted that this study analyzed gene expression and miRNA profiles of human blood samples. Although alterations of apoptotic markers in the schizophrenic brains have been reported [8–12], whether the alterations in apoptosis-related DEGs and DEMs observed in this study are related to the apoptotic activation in the brain remains to be studies. Most RNAs in the blood samples are from PBMCs, but very small amounts of RNAs and RNA fragments could come from the brain through secreted or extracellular vesicles, which might contribute to the blood RNA profiling. It remains to be elucidated whether the blood alterations of apoptosis-related gene expression represent or are related to those in the brain. Nevertheless, our findings of the diagnostic value of the model based on apoptosis-related gene expressions suggest that the apoptosis-related gene expression profile in the blood either corresponds to similar changes in the brain or is somehow involved in the specific brain changes.

Several limitations of our study need to be noted. First, the diagnostic model is only validated in samples from public data, and clinical samples also need to be enrolled to confirm the diagnostic performance of the model. Validation of the results of this study in a new and separate set of clinical data will strengthen the conclusion. Second, ceRNA networks were obtained via a bioinformatics analysis, and whether specific ceRNA regulatory mechanisms exist in patients with schizophrenia needs to be investigated through *in vivo* and *in vitro* experiments. This will be the focus of future research.

## 5. Conclusion

Through analyzing the gene expressions and the lncRNA and miRNA profiles of blood samples from schizophrenic patients and controls, we constructed a robust diagnostic model based on apoptosis-related genes. Our initial study suggests that this model can be used for risk classification of individuals with schizophrenia and potentially has a diagnostic value. Moreover, a lncRNA-related ceRNA network may offer new insight into the regulatory mechanisms of schizophrenia. These findings may help improve the diagnostic efficiency of schizophrenia and provide guidance for personalized management for schizophrenic patients.

## Figures and Tables

**Table 1:** Detailed information for each dataset included in this analysis.

Datasets	Years	Samples	NC	SCZ	Database	Platforms
GSE27383	2013	PBMC	29	43	NCBI GEO	[HG-U133_Plus_2] Affymetrix Human Genome U133 Plus 2.0 Array
GSE38485	2012	Whole blood	22	15	NCBI GEO	Illumina HumanRef-8 v3.0 Expression BeadChip
	2012	Whole blood	96	106	NCBI GEO	Illumina HumanHT-12 V3.0 Expression BeadChip
GSE54913	2014	PBMC	12	18	NCBI GEO	Arraystar Human LncRNA microarray V2.0 (Agilent-033010 Probe Name version)
E-MTAB-3303	2015	PBMC	83	128	ARRAYEXPRESS	A-MEXP-1820—Illumina Human microRNA V2 BeadChip

NC: normal control; PBMCs: peripheral blood mononuclear cells; SCZ: schizophrenia.

## Data Availability

The data used to support the findings of this study are available from the corresponding author upon request.
